# Supplementary material for: Plasmapheresis in the ICU
Source: Medicina (Kaunas). 2023 Dec 12;59(12):2152. doi: 10.3390/medicina59122152 (PMC10744423; doi:10.3390/medicina59122152)
Supplement: Supplementary file 1 [file medicina-59-02152-s001.zip › medicina-2697881-supplementary.pdf]

Supplementary Materials:

Expanded Disability Status Scale (EDSS)

0 = Normal neurologic exam (all grade 0 in Functional Systems [FS]; Cerebral grade 1 acceptable)

1.0 = No disability, minimal signs in one FS (ie, grade 1 excluding Cerebral grade 1)

1.5 = No disability minimal signs in more than one FS (more than one grade 1 excluding Cerebral grade 1)

2.0 = Minimal disability in one FS (one FS grade 2, others 0 or 1)

2.5 = Minimal disability in two FS (two FS grade 2, others 0 or 1)

3.0 = Moderate disability in one FS (one FS grade 3, others 0 or 1), or mild disability in three or four FS (three/four FS grade 2, others 0 or 1) though fully ambulatory

3.5 = Fully ambulatory but with moderate disability in one FS (one grade 3) and one or two FS grade 2; or two FS grade 3; or five FS grade 2 (others 0 or 1)

4.0 = Fully ambulatory without aid, self-sufficient, up and about some 12 hours a day despite relatively severe disability consisting of one FS grade 4 (others 0 or 1, or combinations of lesser grades exceeding limits of previous steps. Able to walk without aid or rest some 500 meters.

4.5 = Fully ambulatory without aid, up and about much of the day, able to work a full day, may otherwise have some limitation of full activity or require minimal assistance; characterized by relatively severe disability, usually consisting of one FS grade 4 (others 0 or 1) or combinations of lesser grades exceeding limits of previous steps. Able to walk without aid or rest for some 300 meters.

5.0 = Ambulatory without aid or rest for about 200 meters; disability severe enough to impair full daily activities (eg, to work full day without special provisions). (Usual FS equivalents are one grade 5 alone, others 0 or 1; or combinations of lesser grades usually exceeding specifications for step 4.0.)

5.5 = Ambulatory without aid or rest for about 100 meters; disability severe enough to preclude full daily activities. (Usual FS equivalents are one grade 5 alone, others 0 or 1; or combinations of lesser grades usually exceeding those for step 4.0.)

6.0 = Intermittent or unilateral constant assistance (cane, crutch, or brace) required to walk about 100 meters with or without resting. (Usual FS equivalents are combinations with more than two FS grade 3+.)

6.5 = Constant bilateral assistance (canes, crutches, or braces) required to walk about 20 meters without resting. (Usual FS equivalents are combinations with more than two FS grade 3+.)

7.0 = Unable to walk beyond about 5 meters even with aid, essentially restricted to wheelchair; wheels self in standard wheelchair and transfers alone; up and about in wheelchair 12 hours a day. (Usual FS equivalents are combinations with more than one FS grade 4+; very rarely, pyramidal grade 5 alone.)

7.5 = Unable to take more than a few steps; restricted to wheelchair; may need aid in transfer; wheels self but cannot carry on in standard wheelchair a full day; may require motorized wheelchair. (Usual FS equivalents are combinations with more than one FS grade 4+.)

8.0 = Essentially restricted to bed or chair or perambulated in wheelchair, but may be out of bed itself much of the day; retains many self-care functions; generally has effective use of arms. (Usual FS equivalents are combinations, generally grade 4+ in several systems.)

8.5 = Essentially restricted to bed much of the day; has some effective use of arm(s); retains some self-care functions. (Usual FS equivalents are combinations, generally 4+ in several systems.)

9.0 = Helpless bed patient; can communicate and eat. (Usual FS equivalents are combinations, mostly grade 4+.)

9.5 = Totally helpless bed patient; unable to communicate effectively or eat/swallow. (Usual FS equivalents are combinations, almost all grade 4+.)

10 = Death due to MS

Neurological Disability Score Evaluation Form

Scoring Instructions:
- **0 for no deficit**
- **1 for mild deficit**
- **2 for moderate deficit**
- **3 for severe deficit**
- **4 for complete absence of function or severest deficit**

Evaluation:
Cranial Nerves:
- **Papilledema: ____**
- **EOM weakness, Cr III: ____**
- **EOM weakness, Cr VI: ____**
- **Face weakness: ____**
- **Palate weakness: ____**
- **Tongue weakness: ____**

Muscle Weakness:
- **Respiratory: ____**
- **Shoulder abduction: ____**
- **Brachioradialis: ____**
- **Extension at elbow: ____**
- **Extension at wrist: ____**
- **Flexion at wrist: ____**
- **Extension of fingers: ____**
- **Flexion of fingers: ____**
- **Intrinsic hand: ____**
- **Iliopsoas: ____**
- **Glutei: ____**
- **Quadriceps: ____**
- **Hamstrings: ____**
- **Dorsiflexors: ____**
- **Plantar flexors: ____**
- **Biceps brachii: ____**

Reflexes:
- **Biceps brachii: ____**
- **Triceps brachii: ____**
- **Brachioradialis: ____**
- **Quadriceps femoris: ____**
- **Triceps surae: ____**

Sensation (Index Finger & Great Toe):

- **Touch-pressure (Index finger): ____**
- **Pricking pain (Index finger): ____**
- **Vibration (Index finger): ____**
- **JP (Index finger): ____**
- **Touch-pressure (Great toe): ____**
- **Pricking pain (Great toe): ____**
- **Vibration (Great toe): ____**
- **JP (Great toe): ____**

Sum Total: ______
